# Postnatal Ontogeny of the Cranial Base and Craniofacial Skeleton in Male C57BL/6J Mice: A Reference Standard for Quantitative Analysis

**DOI:** 10.3389/fphys.2015.00417

**Published:** 2016-01-12

**Authors:** Siddharth R. Vora, Esra D. Camci, Timothy C. Cox

**Affiliations:** ^1^Departments of Oral Health Sciences, University of WashingtonSeattle, WA, USA; ^2^Orthodontics, University of WashingtonSeattle, WA, USA; ^3^Center for Developmental Biology and Regenerative Medicine, Seattle Children's Research InstituteSeattle, WA, USA; ^4^Pediatrics (Craniofacial Medicine), University of WashingtonSeattle, WA, USA; ^5^Department of Anatomy and Developmental Biology, Monash UniversityClayton, VIC, Australia

**Keywords:** cranial base, synchondroses, postnatal growth, craniofacial, morphometrics, mouse models

## Abstract

Growth of the craniofacial skeleton is a complex process controlled by both genetic and epigenetic factors, perturbations of which can lead to varying degrees of dysmorphology. Mouse models that recapitulate clinical craniofacial phenotypes are instrumental in studying the morphogenetic progression of diseases as well as uncovering their genetic and molecular bases. Commonly encountered phenotypes in these models include defects in the cranial base synchondroses, calvarial sutures, mandible or the midface, or any combination thereof, with the concurrent presence of altered overall craniofacial growth. However, the literature lacks an adequate normative timeline of developmental events and growth trends that shape the mouse craniofacial skeleton. In this report, we analyzed the postnatal craniofacial ontogeny (from postnatal day 7 [P7] through to P112) of male mice from the most widely used inbred mouse strain, C57BL/6J, using high-resolution microcomputed tomography (μCT) in combination with classic morphometric approaches. We also evaluated cranial base synchondroses at the histological level, and compared it to μCT-generated data to assess the timing and pattern of closure of these structures. Our data underscore the complex and unique growth patterns of individual bones and cranial regions and highlight the need to include younger animals in studies aimed at analyzing craniofacial growth processes. Furthermore, these data serve as a reference standard for future quantitative work.

## Introduction

Nearly one in four birth defects have craniofacial involvement, with many patients requiring significant orthodontic, orthopedic, or surgical intervention (Posnick and Ruiz, 2000; Cunningham et al., 2007; Saltaji et al., 2014). Animal models have played a pivotal role in understanding the genetic mechanisms and pathways involved in some of these disease processes, particularly through the investigation of mutant phenotypes in mice (Wilkie and Morriss-Kay, 2001; Hallgrímsson and Lieberman, 2008). Despite the overt differences in craniofacial form between mice and humans, similar developmental processes and comparable phenotypic variability support their utilization as models (Hallgrímsson and Lieberman, 2008; Martínez-Abadías et al., 2012). Advantages of studying human disorders in laboratory mouse models (like the C57BL/6J) include their uniform genetic background, the relative ease with which their genome can be modified, and the degree to which their environment can be controlled. This level of experimental control allows investigators to dissect out the individual genetic and epigenetic contributions to phenotypes or disease states. Nevertheless, the value of this model is inherently dependent on a detailed knowledge of the ontogeny of the postnatal mouse cranial and facial skeleton. The Jackson Laboratory (jax.org), the largest supplier of mouse resources for research, provides a set of basic cranioskeletal metrics for various wildtype and mutant strains based on caliper measurements, but more detailed data on the ontogeny of postnatal cranioskeletal growth is lacking.

In the last decade, much research effort has been centered on the cranial vault and specifically the calvarial sutures as their premature fusion, termed craniosynostosis, is a relatively common presentation in the pediatric population (~1 in 2500 live births) and is associated with significant cranioskeletal deformity (Morriss-Kay and Wilkie, 2005). In contrast, relatively little attention has been paid to the bones of the facial skeletal or the cranial base, despite the general acceptance from the historical literature that the cranial base, in particular, has a primary role in driving postnatal cranioskeletal growth and form (Lieberman et al., 2000; Parsons et al., 2015). Indeed, in both patients and in the respective mouse models of the major syndromic forms of craniosynostosis, cranial base, and facial bone abnormalities are evident, in some cases occurring prior to the fusion of the calvaria (Perlyn et al., 2006; Laurita et al., 2011; Purushothaman et al., 2011). In addition, in many mouse models of severe midface hypoplasia (type III malocclusion) and cranial dysmorphology, the cranial base, and/or facial bone growth is abnormal while many of the calvarial sutures can remain largely unaffected. These observations support the notion that changes in regions other than the cranial vault have a significant impact on the morphology of the skull.

The murine cranial base first appears as a cartilaginous anlage, which begins to ossify *in utero* to form elements of the sphenoid and the occipital bones (Chai and Maxson, 2006; McBratney-Owen et al., 2008). Expansion of the cranial base occurs at cartilaginous growth sites called synchondroses that, although similar in function to calvarial and facial sutures, are different in cellular architecture and regulatory processes. Although most of the calvarial sutures do not fuse during the lifespan of mice, the cranial base synchondroses, and facial sutures do fuse and must do so in a predictable manner to attain the characteristic cranioskeletal form that defines each strain. However, strain-specific normative data is not available. In this study, we have employed high-resolution microcomputed tomography (μCT) and histology to characterize in detail the postnatal cranioskeletal ontogeny of male C57BL/6J mice, one of the most commonly used inbred mouse strain. The data show the robustness of postnatal changes in cranioskeletal growth and therefore should serve as a reference standard for future studies using this strain that aim to examine craniofacial disease models as well as for comparative studies using strains of other genetic backgrounds.

## Materials and methods

### Animals

The highly inbred wildtype strain C57BL/6J (stock #000664; Jackson Laboratories, ME) was used for this study. Mice were maintained on a 12 h light cycle in a controlled environment with standard temperature (73 ± 3°F) and relative humidity (~30 ± 5%) levels, at Seattle Children's Research Institute (SCRI; Seattle, WA). All animal procedures were approved by the Institutional Animal Care and Use Committee (IACUC) at SCRI. Breeders were selected at 2 months of age and fed pelleted high fat, low carbohydrate diets optimal for post-partum breeding (Pico® Rodent Diet 20 - 5058^*^). Sires were seperated at P1 and litters were weaned at P21. Post-weaning, animals were fed pelleted stock diet (Pico® Rodent Diet 20 - 5053^*^). Animals were euthanized by CO_2_ inhalation at desired ages (postnatal day P7, P14, P21, P28, P56, P84, P112 for micro-CT analysis and additionally, P76 and P90 for histology). To minimize differences due to sexual dimorphism, only male mice (*n* = 4 in each age group) were used for the quantitative analysis, while male and female mice were used for the qualitative study of synchondroses (*n* = 14–16 in each age group).

### Scanning, reconstruction, and landmark placement

Following euthanasia, crania were imaged using a Skyscan 1076 micro-Computed Tomography (μCT) scanner at 55 kV, 179 μA with a scan resolution of 17.21 μm. All data were reconstructed using Nrecon (Version 1.6.9.4) with consistent thresholding parameters. Reconstructions were converted to 3D volumes and landmarks placed manually on the rendered data using the freeware, Drishti v2.4 (Limaye, 2012). All landmarks were digitized by the same investigator with a set of 21 single and 17 paired landmarks for a total of 55 landmarks (see Supplementary Figure 1, Supplementary Table 1). At P7, the bregma and lambda are not yet ossified (Supplementary Table 1, #13 and #14, respectively) and hence soft tissue points in the vicinity were selected as landmark proxies in these animals by adjusting volume rendering parameters (represented as “Transfer Functions” in Drishti v2.4). The mean intra-observer variability in identifying bregma and lambda were 0.14 and 0.17 mm, respectively, which was higher than that typically found for hard tissue landmarks (Aneja et al., 2015). For the remaining points at P7 and all other ages, the rendering parameters were maintained to ensure consistency and validity of comparisons. We chose not to include animals younger than P7 in our study due to the greater variability in degree of ossification and the inherent decreased reliability of landmark identification as a result.

### Measurements

When analyzing growth in the antero-posterior dimension we calculated projected distances between parallel planes passing through selected landmarks. To achieve this, a best-fit mid-sagittal plane was manually constructed for each scan (Supplementary Figure 2, top), following which a transverse plane was constructed perpendicular to the mid-sagittal plane, touching the palatal cusp tips of the right and left first and second molars (for P7 animals these teeth have not yet erupted but are visible in their crypts). Finally, axial planes passing through the landmarks of interest were constructed perpendicular to the first two planes. For bilaterally paired landmarks, an average of the right and left measurements was used for the analysis An example of this is shown in Supplementary Figure 2 depicting how the palatal, maxillary, and premaxillary dimensions were measured. This method of measurement is appropriate since one of our goals was to assess the growth pattern of regions within the craniofacial skeleton (i.e., the cranial base, vault, and facial skeleton), each comprised of multiple ordered individual bones. Consequently, growth in the A-P dimension can result in dynamically altered architectural arrangement of bones within each region in multiple planes, making interpretation of Euclidian distance difficult. Additionally, our method allows us to appreciate how different regions of the craniofacial skeleton are growing in proportion to the overall A-P dimension. Since the scans of each animal were not always obtained with teeth in occlusion, projected distances could not be reliably applied to the mandible and Euclidian distances were utilized instead. For measurements in the transverse dimension, the Euclidian distance between the right and left paired set of selected landmarks was calculated. Angular measurements were obtained using mid-sagittal landmarks.

To assess significance, Students' *t*-tests (two-tailed, equal variance) were carried out between corresponding measurements at consecutive ages. Hence P14 was compared to P7, P21 was compared to P14 and so forth. It should be noted that significance can be influenced by the age intervals being tested. For example if we tested a larger age interval, differences would typically reach significance. However, with the additional of extra time points within the interval, the differences in growth between intervals become smaller in magnitude and, given the variability and number of animals used in this study, thus formally lose significance.

### Histology

For the purpose of histology, basicrania were isolated from euthanized animals and immediately fixed in 4% paraformaldehyde for 24 h. Following fixation, specimens were decalcified in 14% EDTA (pH 7.4) for 2 weeks (longer decalcification was necessary for older animals, tested by subsequent x-ray scans to ensure complete removal of mineral). Decalcified tissues were then serially dehydrated using ethanol and embedded in paraffin. Eight micron thick sections were cut in the sagittal plane, dewaxed, hydrated, and stained with hematoxylin-eosin or Safranin O-fast green.

## Results

### Postnatal craniofacial skeletal growth in the C57BL/6J mouse

Postnatal growth of the C57BL/6J craniofacial skeleton was quantified by measuring changes in length along the transverse, vertical, and antero-posterior (A-P) planes as well as angular changes in the mid-sagittal plane in animals at P7, P14, P21, P28, P56, P84, and P112. While most studies identify the cranial base, the cranial vault, and the face as morphologically semi-independent modules, it has been shown that the cranial vault and base behave developmentally as one integrated complex (Lieberman et al., 2000; Hallgrímsson et al., 2007). Hence we analyzed growth changes in the facial region and a combined cranial region (vault + base).

**Figure 1:**
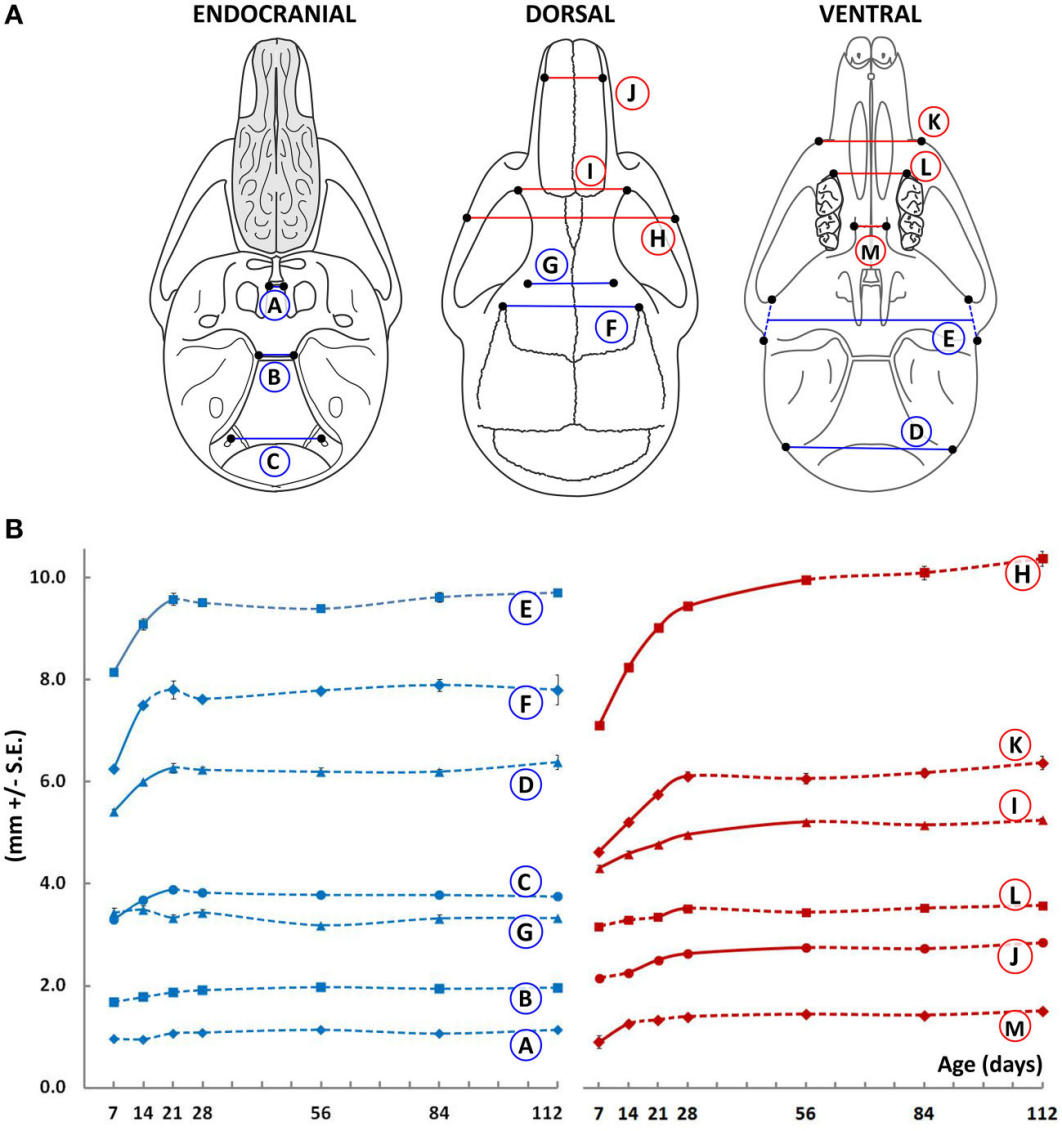
**Transverse growth of the mouse craniofacial skeleton**. **(A)**Schematic of the dorsal, ventral, and endocranial views of the mouse skull depicting paired lateral landmarks (black dots) used to obtain measurements in the cranial (blue) and facial (red) regions. **(B)** Growth curves showing changes in the transverse measurements (mm ± S.E., y-axis) with age (postnatal day, x-axis). The label adjacent to each curve is depicted in the schematic shown (see Table 1 for description). Solid and dashed lines between data points represent significant and non-significant changes, respectively, for that interval (Students' *t*-test, *p* < 0.05).

The width of the basisphenoid was measured at the rostral and caudal borders of its dorsal surface, corresponding to the intersphenoidal synchondrosis (ISS) and spheno-occipital synchondrosis (SOS), respectively (Figure 1, circled A and B, respectively). While there is incremental growth with age after P7, the increases only reach statistical significance when analyzed over the duration of time points investigated. When P112 animals are compared directly to P7 animals, the width of the basisphenoid at its caudal border is slightly greater (by 0.25 mm, *p* = 0.028), while its rostral width does not change. Width of the cranial base measured at the hypoglossal canal increases significantly between P7 and P21, plateauing thereafter (Figure 1, circled C). The width of the cranial vault measured at the inferior (near the paraoccipital process) and mid-vertical aspects (zygomatic root of temporal bone) increases significantly from P7 to P14 and plateaus thereafter (Figure 1, circled D and E, respectively). Notably, the antero-superior portion of the cranial vault does not widen appreciably beyond P14 (Figure 1, circled F and G, respectively). Together, these data suggest that the width of the cranial region (Figure 1, blue lines) is established early in ontogeny with little change occurring after 3 weeks of age. In comparison, the width of the facial region continues to increases, albeit modestly, even after growth of the cranium has declined (Figure 1, red lines). Inter-zygomatic width increases significantly beyond P28, as do the inter-orbital and inter-maxillary widths (Figure 1, circled H, I, and K, respectively). It should be noted however that centrally located structures in the facial region do not follow this pattern. For instance, the inter-molar and inter-palatal width does not change appreciably beyond P14 (Figure 1, circled L and M, respectively), while other more lateral structures continue to widen (for example, Figure 1, circled H, I, J). These data are in line with human studies (Edwards et al., 2007), which suggest that transverse dimensions of the craniofacial skeleton are established early in ontogeny.

**Table 1:** **List of measurements used in the study with corresponding landmarks used to analyze individual bones, cranial regions, and angles**.

**Label**	**Bone/region**	**Landmarks**	**Type**
A	Basisphenoid (rostral)	R-L 29	Transverse
B	Basisphenoid (caudal)	R-L 30	Transverse
C	Cranial base (posterior region)	R-L 32	Transverse
D	Inter-mastoid width	R-L 17	Transverse
E	Inter-zygomatic root width	R-L (10/11)	Transverse
F	Anterior cranial vault width	R-L 12	Transverse
G	Interior frontal arch width	R-L 34	Transverse
H	Inter- zygomatic arch width	R-L 8	Transverse
I	Inter-orbital width	R-L 6	Transverse
J	Anterior nasal width	R-L 4	Transverse
K	Inter-maxillary width	R-L 7	Transverse
L	Inter-molar width	R-L 23	Transverse
M	Palatal width	R-L 21	Transverse
N	Posterior cranial vault	31-15	Vertical
O	Middle cranial vault	28-14	Vertical
P	Anterior cranial vault	27-13	Vertical
Q	Frontal crest height	26-33	Vertical
R	Anterior nasal height	2-3	Vertical
S	Facial height	19-5	Vertical
T	Posterior nasal height	20-26	Vertical
U	Anterior pharyngeal height	22-27	Vertical
V	Posterior pharyngeal height	24-28	Vertical
W	Total skull length	1-16	Antero-Posterior
X	Cranial vault length	25-16	Antero-Posterior
Y	Cranial base (rostral)	26-31	Antero-Posterior
Z	Anterior cranial base	25-26	Antero-Posterior
AA	Basiocciput	28-31	Antero-Posterior
BB	Basisphenoid	27-28	Antero-Posterior
CC	Presphenoid	26-27	Antero-Posterior
DD	Facial region length	1-22	Antero-Posterior
EE	Palate	22-20	Antero-Posterior
FF	Maxilla	20-18	Antero-Posterior
GG	Premaxilla	18-1	Antero-Posterior
HH	Nasal	3-5	Antero-Posterior
II	Zygomatic	8-9	Antero-Posterior
JJ	Mandibular posterior height	35-36	Vertical
KK	Mandibular length (superior)	35-38	Antero-Posterior
LL	Mandibular length (inferior)	36-37	Antero-Posterior
MM	Upper Jaw	1-(10/11)	Antero-Posterior
NN	Cranial base angle	31-27-25	Angle
OO	Anterior cranial vault angle	27-25-13	Angle
PP	Mid-anterior cranial vault angle	25-13-14	Angle
QQ	Mid-posterior cranial vault angle	13-14-15	Angle
RR	Posterior cranial vault angle	14-15-16	Angle
SS	Snout angle	27-25-2	Angle
TT	Facial angle	22-5-2	Angle

*Labels (column 1) are annotated in schematics in Figures 1–**7**; Landmarks (column 3) are depicted in Supplementary Figure 1 and described in Supplementary Table 1; R, right; L, left*.

**Figure 2:**
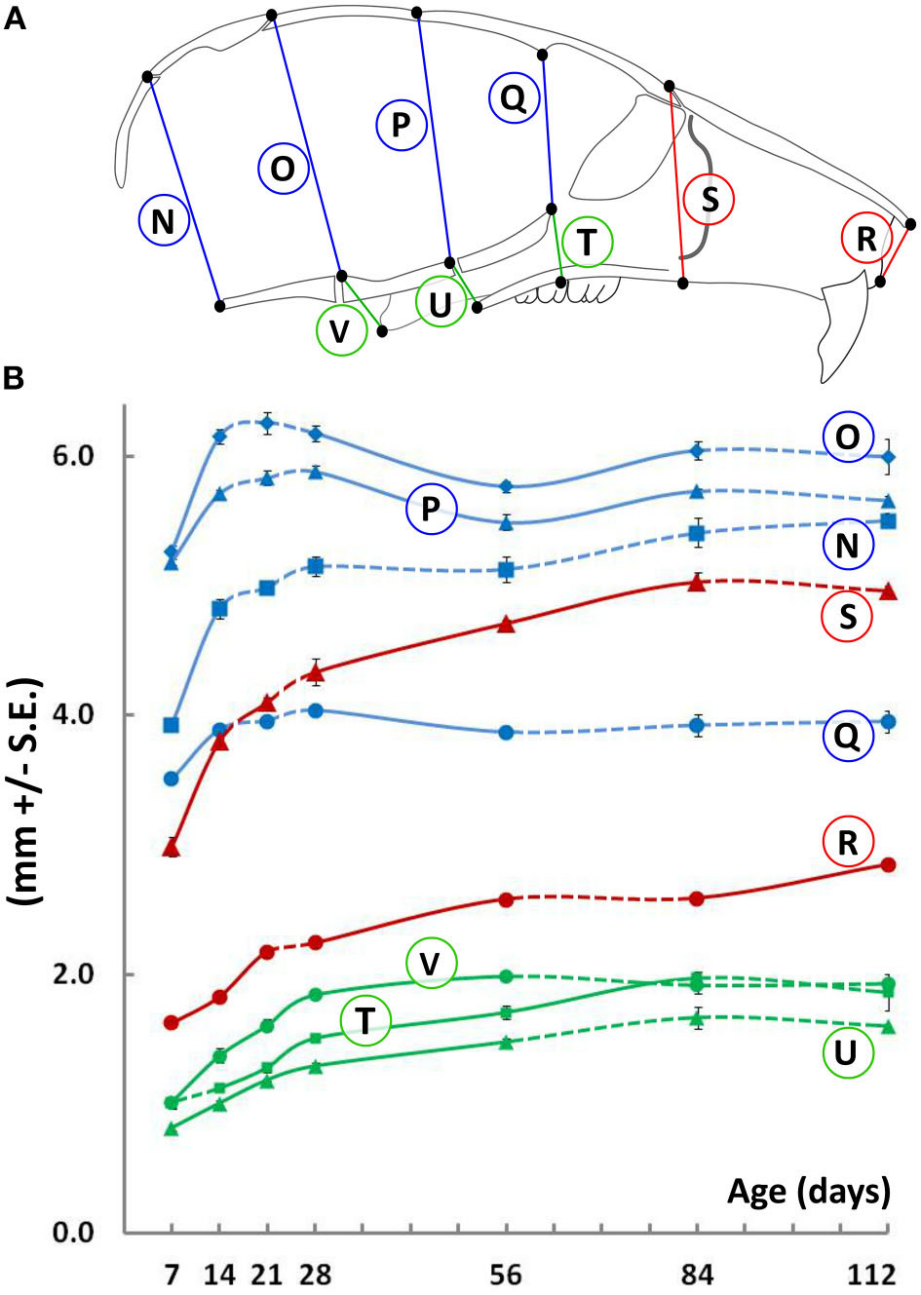
**Vertical growth of the mouse craniofacial skeleton**. **(A)** Schematic of the mid-sagittal view of the mouse skull depicting landmarks (black dots) used to obtain vertical measurements in the cranial (blue) and facial (red) and pharyngeal (green) regions. **(B)** Growth curves showing changes in vertical measurements (mm ± S.E., y-axis) with age (postnatal day, x-axis). The label adjacent to each curve is depicted in the schematic shown (see Table 1 for description). Solid and dashed lines between data points represent significant and non-significant changes, respectively, for that interval (Students' *t*-test, *p* < 0.05).

Vertical measurements were made using medial (mid-sagittal) landmarks in the cranial and facial regions (Figure 2). Similar to width, the height of the cranial vault increases significantly between P7 and P14 (Figure 2, blue lines), following which changes are smaller in magnitude. In contrast, the height of the facial region increases significantly beyond P21 in both the anterior (nasal, Figure 2, red lines) and posterior (pharyngeal, Figure 2, green lines) areas. Curiously, between P28 and P56, there is a small yet significant decrease in cranial vault height when measured at the bregma-ISS and lambda-SOS landmarks (Figure 2, circled P and O, respectively). However, this decrease is coupled with an increase in the posterior nasal and pharyngeal heights, maintaining overall vertical dimensions (Figure 2, circled T, U, and V).

**Figure 3:**
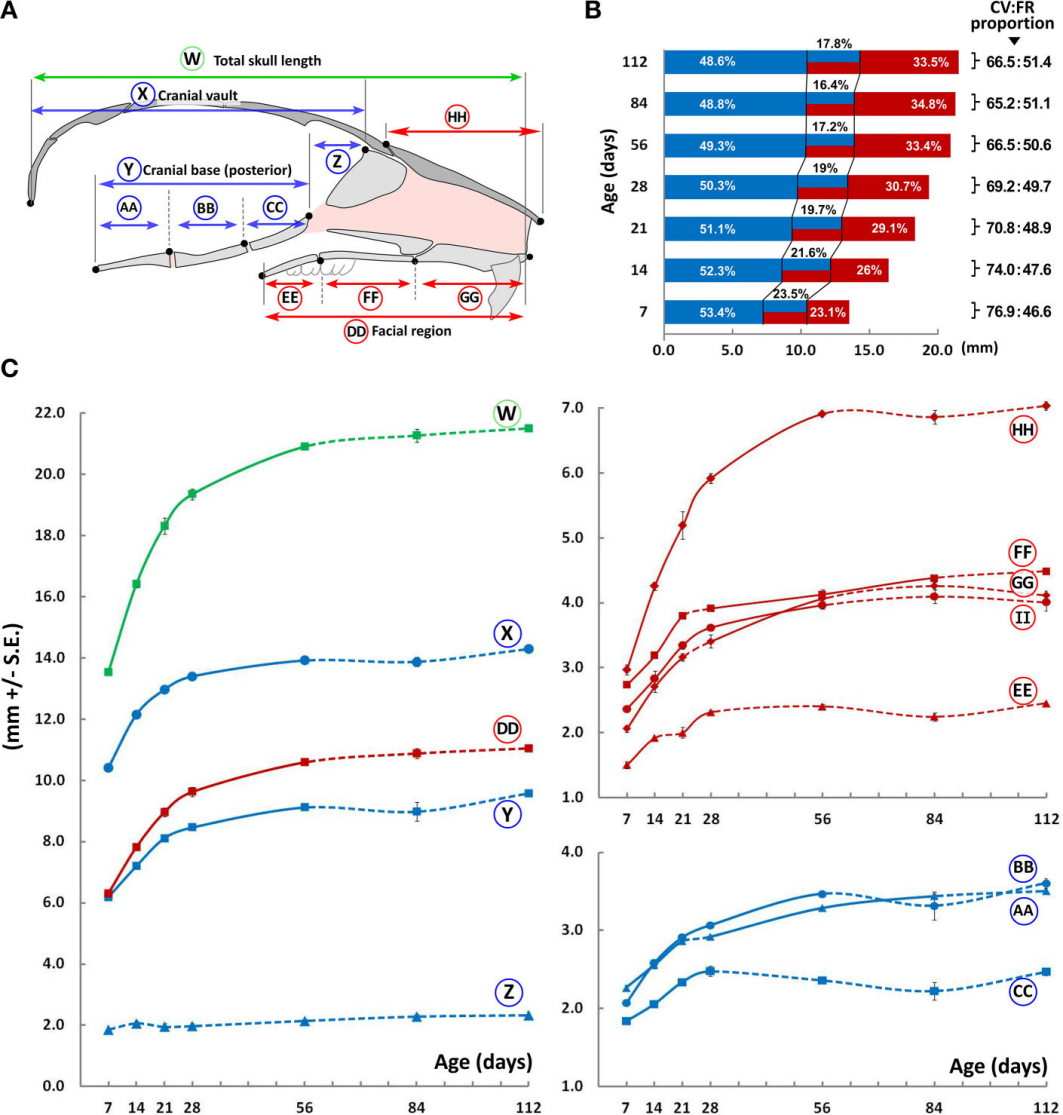
**Antero-posterior growth of the mouse craniofacial skeleton**. **(A)** Schematic of the mid-sagittal structures of the mouse skull depicting landmarks (black dots) and planes (vertical lines) used to obtain measurements in the cranial (blue) and facial (red) regions (see Methodology, Figure 1). **(B)** Bar graph depicting growth of the cranial vault (blue) and facial region (red) as well as the overlap (blue/red) between them (see **A** for schematic of each region). Numbers next to square parenthesis indicate the proportions of the entire A-P dimension of the skull occupied by the cranial vault and facial regions, inclusive of the overlap between them (CV:FR proportion); while numbers within the bar graph indicate the proportion (percentage) of each region adjusted for overlap (i.e., cranial vault, overlap, and facial regions). **(C)** Growth curves showing changes in A-P measurements of individual bones or facial regions (mm ± S.E., y-axis) with age (postnatal day, x-axis). The label adjacent to each curve is depicted in the schematic shown here and in Figure 5A (see Table 1 for description). Solid and dashed lines between data points represent significant and non-significant changes, respectively, for that interval (Students' *t*-test, *p* < 0.05).

**Figure 4:**
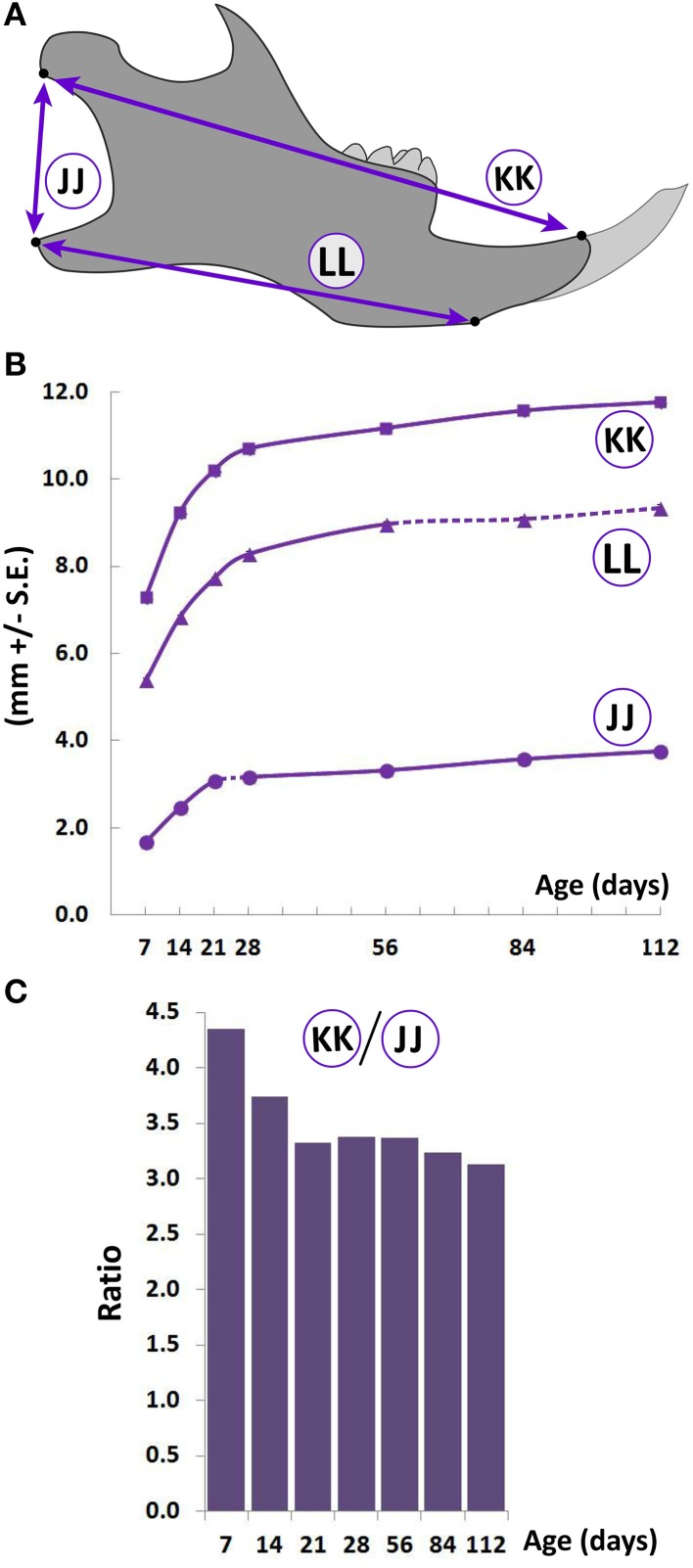
**Postnatal growth of the lower jaw**. **(A)** Schematic of the lateral view of the mouse hemimandible (right) depicting landmarks (black dots) used to obtain measurements. **(B)** Growth curves showing changes in vertical and A-P measurements (mm ± S.E., y-axis) with age (postnatal day, x-axis). The label adjacent to each curve is depicted in the schematic (see Table 1 for description). Solid and dashed lines between data points represent significant and non-significant changes, respectively, for that interval (Students' *t*-test, *p* < 0.05). **(C)** Postnatal change in ratio (y-axis) of the A-P (length, circled KK), and vertical (height, circled JJ) mandibular measurements with age (postnatal day, x-axis).

The overall A-P length of the C57BL/6J mouse skull, as well as the contained cranial and facial regions, increase up to P56 (Figure 3, circled W, X, and DD, respectively), beyond which no significant differences are noted. Within the cranial vault, there is no perceivable increase in length in the anterior part of the cranial base beyond P7 (Figure 3, circled Z), while the posterior portion of the cranial base increases up to P56 (Figure 3, circled Y). When analyzing individual bones of the posterior cranial base, the presphenoid increases rapidly in size up to P28 after which it plateaus (Figure 3, circled CC), while both the basisphenoid and basioccipital increase in length until P56 and P84, respectively (Figure 3, circled BB, and AA, respectively). Within the facial region, the premaxilla, maxilla (Figure 3, circled GG and FF, respectively) and zygomatic bones (Figure 4, circled II) increase rapidly in length until P56, while growth of the palatal bones tends to slow beyond P28 (Figure 3, circled EE).

### Postnatal lower jaw (mandible) growth in the C57BL/6J mouse

As with cranial and facial regions, A-P growth of the mandible is rapid between P7 and P28, reducing thereafter (Figure 4B, circled KK and LL). Vertical mandibular dimensions in the posterior ramus area also increase linearly from P7 to P21, slowing down thereafter (Figure 4B, circled JJ). The ratio between the A-P and vertical measurement decreases from P7 to P21, beyond which the ratio remains relatively stable (Figure 4C), indicating that the rate of vertical growth is larger than that of A-P growth during this early period (i.e., P7-P21) possibly because significant A-P growth has occurred prior to P7.

### Proportional growth changes of the craniofacial skeleton through postnatal ontogeny

It should be noted that the entire palatal bone(s) as well as part of the ethmoid, presphenoid, frontal, maxillary, vomer, and zygomatic bone(s) are contained in an area which overlap both the facial and cranial vault regions. The use of plane-projected distances (see Materials and Methods, Supplementary Figure 2) allowed us to analyze the proportion of the total A-P dimension contained within each of these areas (facial, cranial, and overlap; Figure 3, bar graph). At P7, the cranial region (including the overlap) constitutes ~76.9% of the total A-P dimension, gradually decreasing to ~66.5% by P56. In contrast, the facial region (including the overlap) constitutes ~46.6% of the total A-P dimension at P7, increasing to ~50.6% by P56. Therefore, although both the cranial and facial regions increase in size significantly up to P56, the contribution of the facial region to the overall A-P dimension increases (Figure 3B, red), while that of the cranial region decreases (Figure 3B, blue). Concurrently, the proportional overlap between these regions also reduces with age (from ~23.5 to 17.2%; Figure 3B, blue-red).

**Figure 5:**
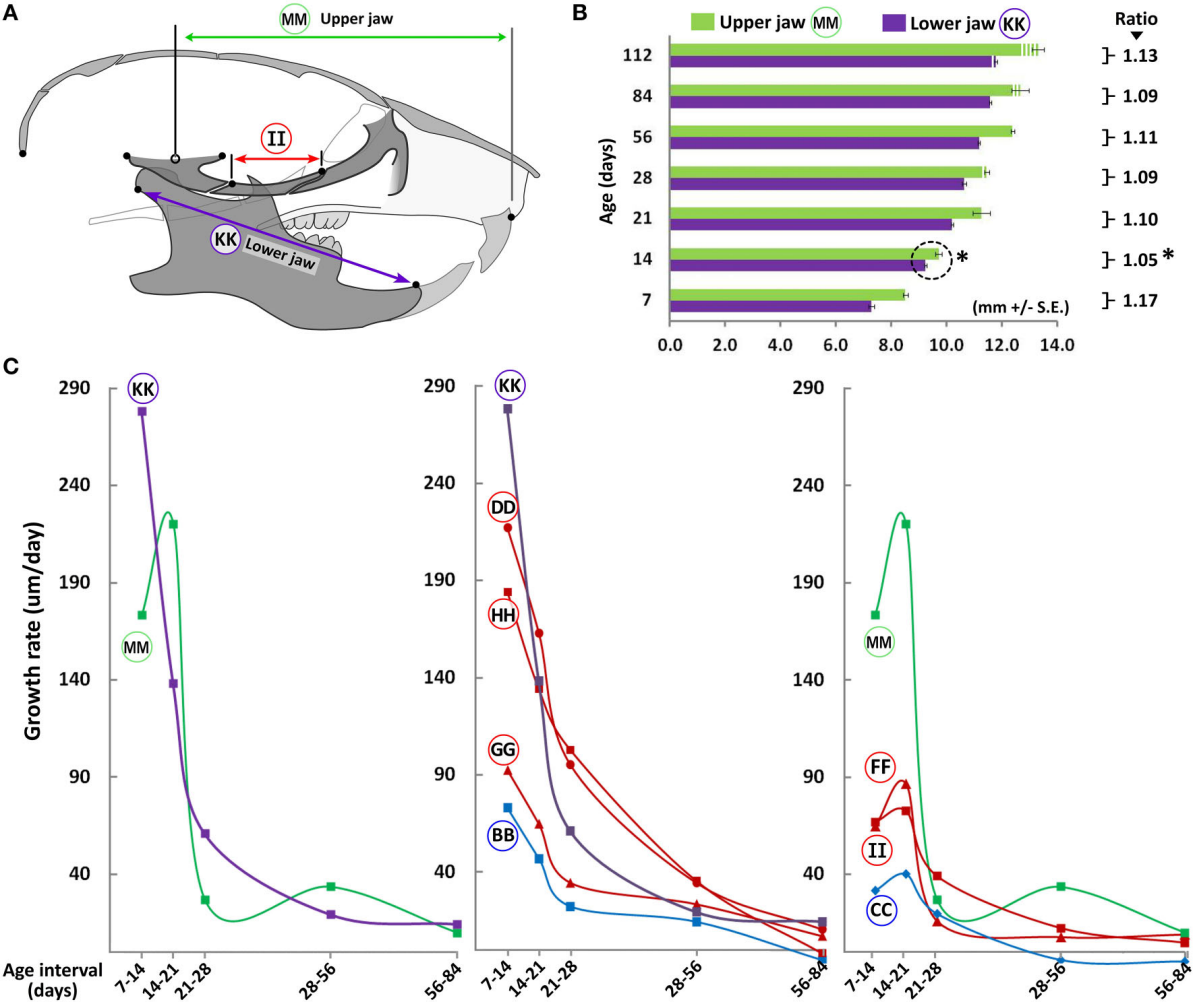
**Coordinated growth of the upper and lower jaws through ontogeny**. **(A)** Schematic of the lateral view of the mouse skull depicting landmarks used to obtain measurements of the upper and lower jaws (mandible). **(B)** Bar graph showing upper (green) and lower (purple) jaw growth with increasing postnatal age. Numbers next to square parenthesis indicate the ratio of the upper/lower jaw at each age, which is at its minimum at P14 (asterisk). **(C)** Growth velocity curves of individual bones or facial regions (μm/day, y-axis) with age interval (x-axis). Label adjacent to each curve is depicted in the schematic shown here and in Figure 3A (see Table 1 for description).

An important consideration when analyzing postnatal A-P changes is the coordinated growth of the upper and lower jaws. The lower jaw is comprised of a pair of bones (hemi-mandibles), each of which articulates with the glenoid fossa at the temporomandibular joint. Because the glenoid fossa is not morphologically well defined in the mouse, we considered the midpoint of the zygomatic process of the temporal bone(s) as the point of articulation. We measured the upper jaw as the distance from this point to a midpoint between the upper incisors (projected distances between constructed planes, as described in Materials and Methods). Both the upper and lower jaws maintain growth until P56 (Figure 5B), slowing thereafter. However, from P7 to P14, a slight increase in relative growth of the lower jaw compared to the upper jaw is noted. This is evident when analyzing the ratio of the upper/lower jaw through postnatal ontogeny (Figure 5B). While this ratio is ~1.17 at P7, it drops significantly to ~1.05 by P14, indicating proportionally greater mandibular growth during this time interval. However, by P21 the upper jaw regains its larger comparative proportion, which is maintained thereafter.

When analyzing growth velocity (μm/day) of specific bones and regions, most cranial and facial bones (including the lower jaw) show a dramatic drop in growth velocity between P14 and P21, reaching near zero values by P84 (Figure 5C, middle panel). However, the upper jaw shows a different trend in that growth velocity peaks from P14 to P21, falling thereafter (Figure 5C, circled MM). This peak seems to trail the timing at which the ratio between the upper and lower jaws is at its lowest (P14, Figure 5B) and its occurrence likely permits the upper jaw to regain its larger proportion compared to the lower jaw. Notably, amongst the individual bones within the anatomical region spanning the upper jaw (facial and cranial), only the maxilla, zygomatic and presphenoid bones display such a cyclic growth pattern (Figure 5C, circled FF, II, and CC, respectively), suggesting growth of these bones are the major contributors to overall upper jaw growth during these stages.

**Figure 6:**
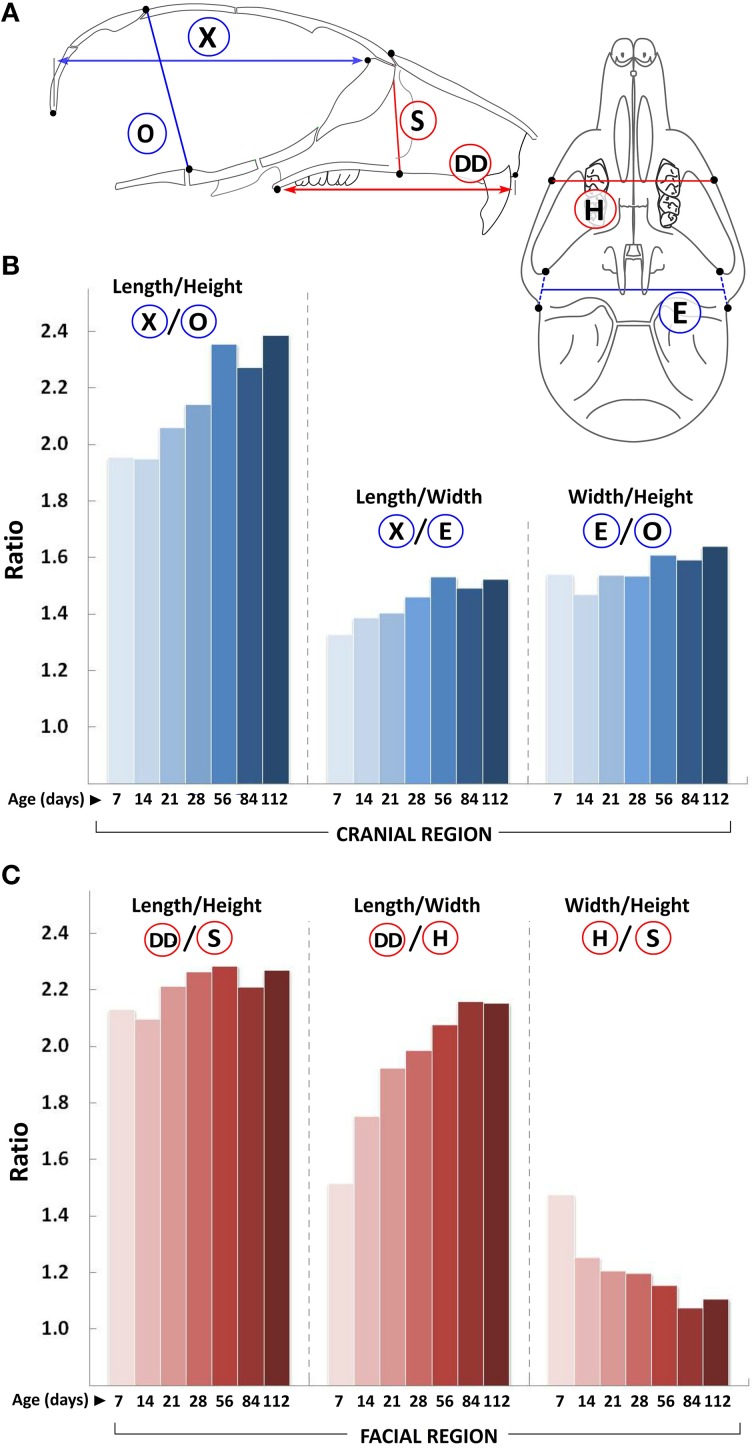
**Changes in craniofacial proportions with growth**. **(A)** Schematic of the mouse skull depicting landmarks (black dots) used to obtain linear measurements. Graphs show ratio of changes in the A-P (length), transverse (width), and vertical (height) planes (y-axis) with age (postnatal day, x-axis) for the cranial **(B)** and facial **(C)** regions. Labels for each set of ratios is depicted in the schematic shown (see Table 1 for description).

In line with human data (Bastir and Rosas, 2006), we find that growth in the facial region continues in all three dimensions after growth in the cranial region has declined, and that growth in the A-P dimension continues well after transverse and vertical growth has plateaued (Figures 1–5). For instance the overall A-P growth (Figure 3, circled W) in just 2 weeks (~2.8 mm from P7 to P21) is similar to the maximal change observed in the vertical dimension (~2.3 mm) and more than half the maximal change observed in the transverse dimension (~4.2 mm). Consequently, the length/height and length/width ratios in both these regions are always >1 (Figure 6). Since growth of the cranial vault in both transverse and vertical planes ceases around P14, the width/height ratio in this region remains relatively unchanged during postnatal growth. However, in the facial region, this ratio progressively decreases with age, indicating a proportionally greater vertical growth as compared to transverse growth. Hence, in the cranial region, the order of larger dimensional magnitude is length > width > height, while in the facial region, this order is length > height > width (Figures 6B,C, respectively).

### Angular changes of the craniofacial skeleton through postnatal ontogeny

**Figure 7:**
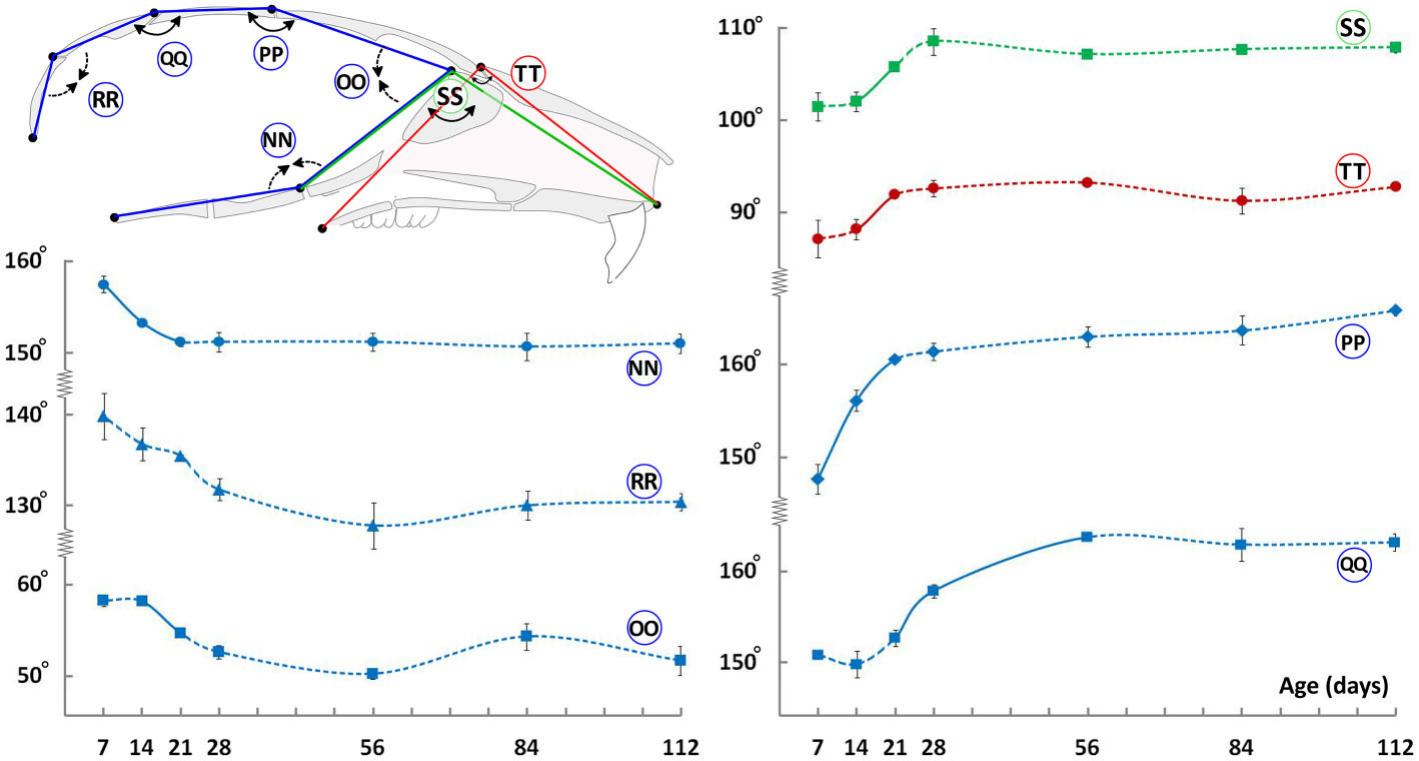
**Angular changes during craniofacial growth**. Schematic (top-left) of the mid-sagittal view of the mouse skull depicting landmarks (black dots) used to obtain angular measurements. Plots show changes in angular measurements (degrees ± S.E., y-axis) with age (postnatal day, x-axis). Label adjacent to each curve is depicted in the schematic shown (see Table 1 for description). Solid and dashed lines between data points represent significant and non-significant changes, respectively, for that interval (Students' *t*-test, *p* < 0.05).

We also evaluated changes in angles between landmarks located on the mid-sagittal plane to assess shape changes through ontogeny. The cranial vault shows a reduction in doming with age as evidenced by an increase in the mid-cranial vault angles (Figure 7, circled PP and QQ), with a concurrent decrease in the anterior and posterior cranial vault angles (Figure 7, circled OO and RR, respectively). These observations along with the vertical changes (Figure 2B), particularly in the facial region, are in line with the perceived flattening of the craniofacial skeleton with age. The cranial base angle decreases from P7 to P21 and plateaus thereafter (Figure 7, circled NN). This postnatal retroflexion of the cranial base is opposite to the increased flexion seen in humans and non-human primates (Ross and Ravosa, 1993; Lieberman and McCarthy, 1999). The facial region shows the opposite trend in that the angles increase from P7 to P21, before plateauing (Figure 7, circled SS and TT respectively). Minimal changes in the facial angles after P28 are consistent with the observed maintenance of the length/height ratio within the facial region (Figure 6C) while vertical and A-P facial dimensions continue to increase (Figures 2, 3, red measurements).

### Timing and pattern of fusion of cranial base synchondroses

Calvaria of P7, P14, P21, P28, P56, P84, and P112 C57BL/6J mice were evaluated using μCT to assess the patency of two paired lateral synchondroses: the alar-alisphenoid (A-ASS), and exoccipital-basioccipital (EO-BOS) synchondroses, and two midline synchondroses: the ISS and SOS.

**Figure 8:**
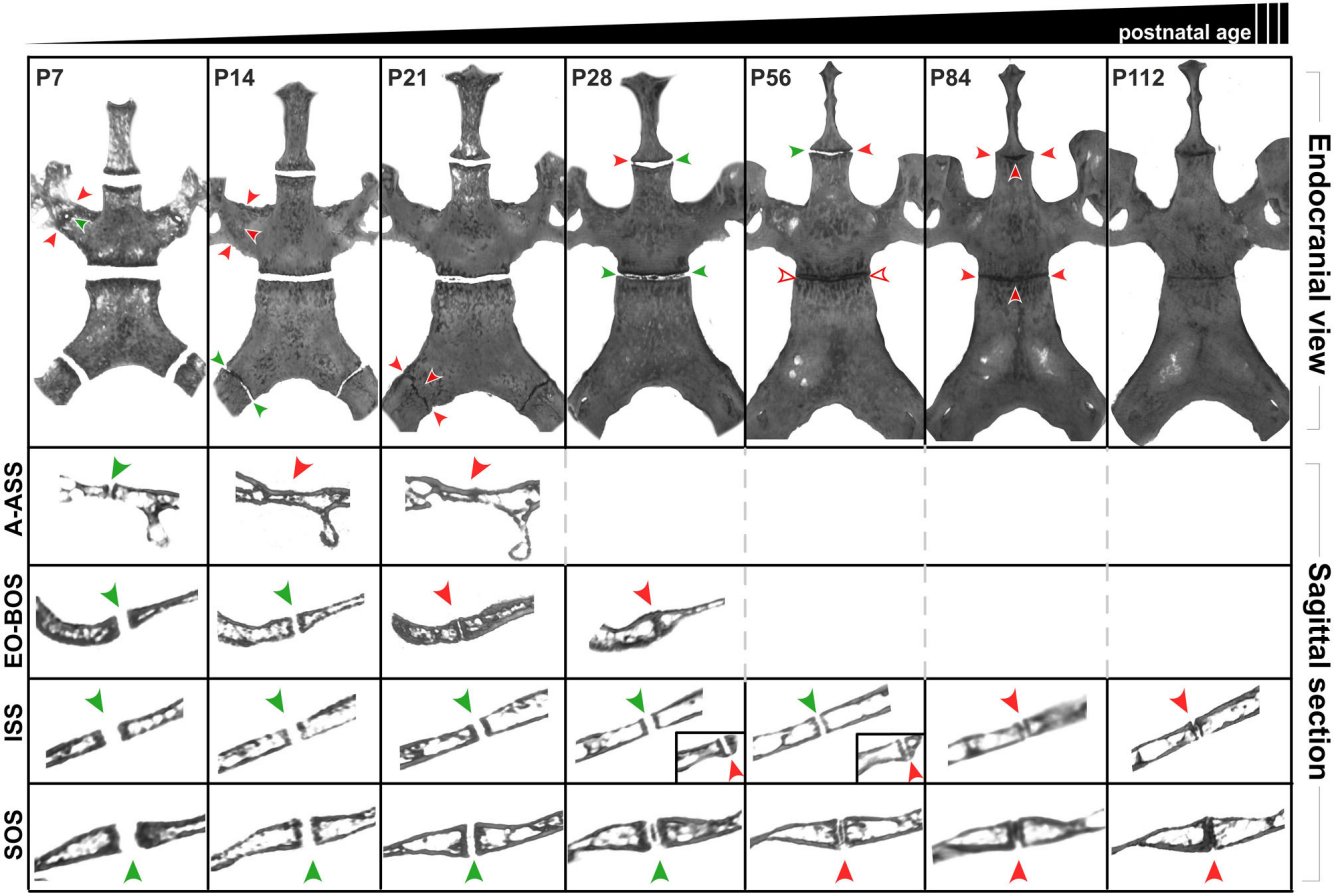
**Postnatal ontogenic changes in the cranial base in C56BL/6J mice**. Top row: Endocranial view of representative reconstructed micro-CT scans of P7, P14, P21, P28, P58, P84, and P112 mouse skulls (left to right). Surrounding structures have been digitally cropped for better visualization of the synchondroses of interest. Green arrowheads indicate areas that appear patent while red arrowheads indicate areas where the synchondroses appear closed. Rows two and three shows sagittal sections perpendicular to the transverse width of the ala-alisphenoid (A-ASS), exoccipital-basioccipital (EO-BOS) synchondroses respectively. Rows four and five shows mid-sagittal sections through the intersphenoidal (ISS) and sphenoccipital synchondroses (SOS) respectively. Insets (Row four, P28 and P56) show sagittal sections of the ISS at the lateral end where it appears to be closing (red arrowheads).

By P7, the A-ASS already appears to be closed at its caudal and rostral ends in 100% of the animals, while the central region is still patent (Figure 8). By P14, this paired synchondrosis is completely closed in all animals. The EO-BOS is wide open in P7 mice, but is reduced in width by P14, as its medial and lateral bony margins approach one another. By P21, areas of fusion can be found along the endo-cranial length of these synchondroses in ~87.5% of animals and by P28, there is near-complete fusion at both endo- and ecto-cranial surfaces in all animals. For most animals, the right and left sides of these paired synchondroses display symmetrical extents and timing of fusion. It is interesting to note that the early fusion found at the A-ASS and EO-BOS coincides with the observed overall decline in transverse cranial growth after P21 (Figure 1).

The ISS remains patent up to P21, however, by this age, small bony outgrowths extending from the ecto-cranial margins of the pre- and basisphenoid bones can be seen laterally in a few animals (~12.5%) while the remaining area of the synchondroses appears patent. By P28, these extensions are larger and appear to completely bridge the ISS (rostro-caudally) in ~26.7% animals (Figure 8). By P56, all animals display a pronounced lateral bridging between the caudal and rostral margins. The SOS remains patent up to P21, however, small radiopaque areas can be seen within the central region of the SOS in ~87.5% animals. By P28, 100% of the animals display radiopaque areas within the SOS, however these regions are not continuous with the corresponding margins of the basisphenoid and basioccipital bones (Figure 8, sagittal views). Beyond P56, these regions appear more condensed, spanning the transverse width of the SOS and by P84, this synchondrosis appears completely radiopaque both in the endocranial and sagittal views (Figure 8).

**Figure 9:**
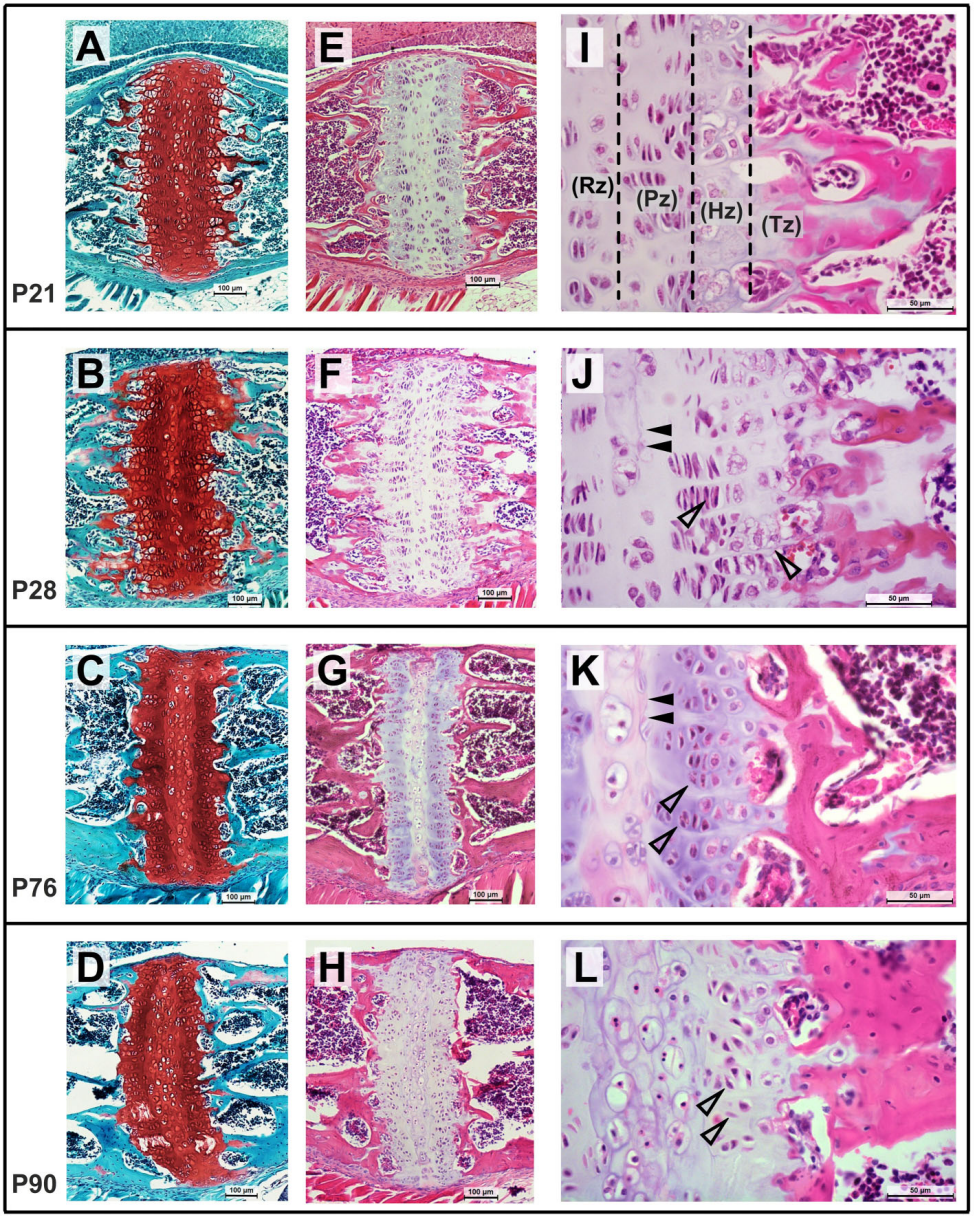
**Postnatal morphological changes in the mouse spheno-occipital synchondroses**. Representative sagittal sections through the SOS at P21 **(A,E,I)**, P28 **(B,F,J)**, P76 **(C,G,K)**, and P90 **(D,H,L)**, stained using Safranin-O, fast green (**A–D**, 10x magnification) and hematoxylin, eosin (**E–H** at10x magnification and **I–K** at 40x magnification). At P21, chondrocytes are arranged in distinct resting (Rz), proliferative (Pz), hypertrophic (Hz), and transitional (Tz) zones, which become less distinct with age **(I,K,L)**. At P28 and P56, some cells in the central resting zone enlarge and show morphological changes with a distinct matrix surrounding them (**J,K**, filled arrowheads). Cells in the proliferative zone at P76 are less flattened and are loosing orientation to the long axis of the synchondroses by P90 (**K,L**, open arrowheads). Fewer hypertrophic cells are visible by P76 and the mineralizing bone in the transitional zone can be seen appearing thicker and more mature.

The ISS and SOS were also studied histologically at select ages to evaluate their cellular morphology and architecture. At P21, chondrocytes within the SOS can be seen arranged in the resting (Rz), proliferative (Pz), hypertrophic (Hz), and transitional (Tz) zones where osteoblasts are actively replacing cartilage with bone (Figure 9I). By P28, some cells within the resting zone begin to show morphological changes characterized by enlarged cytoplasm and condensing nuclei, resembling hypertrophic chondrocytes. The matrix surrounding these cells appears to be separated from the existent matrix by a distinct margin (Figure 9J, filled arrowheads). The locations of the sections were approximated to the μCT scans, revealing that these central areas of altered cellular morphology correspond to the radiopaque regions seen in the scan images (Figure 8). The matrix in this central region takes up the Safranin-O stain with a slightly different intensity, with a distinct margin separating the two as described earlier (Figures 9B–D). The lack of bony tissue suggests that the chondrocyte matrix is mineralizing as opposed to ossifying (i.e., being replaced by bone). By P76, the margin separating the matrix of the central hypertrophic/resting cells and the adjacent proliferative cells is more distinct (Figure 9K, filled arrowheads). Within the proliferative zone, the cells appear stacked with their long axes parallel to the ossifying front as expected, however, they appear less flattened compared to their morphology at P28 (Figures 9J,K, open arrowheads). Notably, the hypertrophic zones at both the margins are considerably smaller than previous time points and the bony margin at the transitional zone appears more condensed, resembling mature lamellar bone. By P90, the central zone appears to be a collection of enlarged, senescent chondrocytes surrounded by mineralized matrix (Figure 9L). The cells that populate the proliferative zone have lost their stacked arrangement and ordered orientation (Figure 9L, open arrowheads). Moreover, a transitional zone cannot be distinguished and few cells resembling hypertrophic chondrocytes are identifiable. The rostral and caudal bony margins appear continuous and significantly thicker than they did at earlier ages (Figures 9F,G,H).

**Figure 10:**
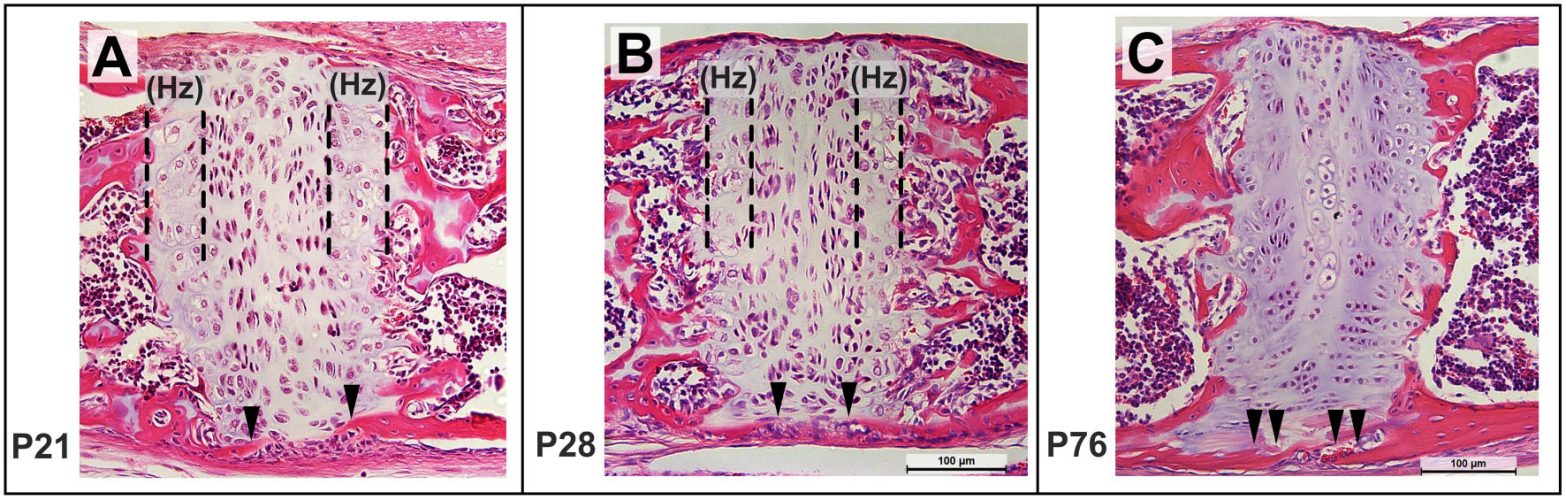
**Postnatal morphological changes in the mouse intersphenoidal synchondroses**. Representative sagittal sections through the ISS at P21 **(A)**, P28 **(B)**, P76 **(C)** stained using hematoxylin, eosin **(**20x magnification). The hypertrophic (Hz) zone appears narrower by P28, and by P76 few hypertrophic cells can be distinguished. Mineralized tissue from the ectocranial surface of the caudal and rostral margins can be seen extending toward each other from P21 to P28 (filled arrowheads) and have bridged the ISS by P76.

Sagittal sections through the ISS at P21 shows the small bony extensions on the ecto-cranial surface (Figure 10A, filled arrowheads), which approach each other by P28 (Figure 10B, filled arrowheads), corresponding to the bridging seen in the μCT scans (Figure 8). While the chondrocytes in the ISS maintain their expected arrangement in the four zones, the hypertrophic zone (Hz) appears smaller at P28 compared to that at P21 (Figures 10B,A, respectively). By P76, the bony bridge is thicker (Figure 10C, filled arrowheads) and the cells within the resting zone of the ISS begin to resemble those in the mineralized, central zone of the SOS (Figure 9L). Once again, a hypertrophic or transitional zone is not easily distinguishable by this age and the rostral and caudal bony margins appear thicker and well defined (Figure 10C).

On the μCT scans, the sagittal views of the ISS and SOS at P84 suggest bony bridging throughout their transverse length (Figure 8). However, we did not find tissue resembling mature bone bridging these structures in our histological sections (Figures 9, 10). Only in some areas of the lateral regions of the ISS can a thin, continuous band of bone matrix be seen traversing rostral and caudal margins (data not shown). As noted above, this suggests that the radiopaque regions seen at these ages are mineralizing cartilage, as opposed to ossifying tissue. Since we decalcify our tissues to facilitate sectioning, we are unable to ascertain the extent of mineralization (i.e., through all the zones) within the synchondroses. It is possible that as the animal ages (beyond P90), the mineralized areas within the synchondroses will eventually be replaced by bone matrix (i.e., ossify) however this cannot be confirmed by examining μCT scans alone.

## Discussion

Geometric morphometrics-based methods are often utilized when analyzing and describing gross craniofacial shape changes. However, they are typically insensitive to smaller, more localized changes as differences between landmark configurations are equilibrated over the entire morphospace. Because we wished to relate precise local and regional measurements to global changes within the skull, we chose to use traditional morphometric methodologies This approach also allowed us to compare postnatal cranioskeletal growth to morphological changes in the cranial base synchondroses (via μCT and histology). Only male mice were used for growth analysis, however, gender specific growth differences do exist. For example P28 male and female mice show small yet significant differences in select width measurements in the facial region as well as some angular measurements (data not shown).

In the age range that we studied here, it is notable that 50% of the total A-P dimension is reached before P7. This is true also for the cranial and facial regions as well as most of the individual bones (Figure 3C). Close to 80% of the growth is completed between P7 and P14 in the cranial region and between P14 and P21 in facial region. Hence, if the aims of a research study were to analyze specific craniofacial growth processes, it would be prudent to include animals younger than P21 to ensure that an active growth period is represented. Using older animals to describe craniofacial phenotype following genetic, epigenetic or environmental perturbations would be analyzing only the cumulative effects of the said perturbation through postnatal ontogeny.

The central radiopaque regions in the SOS that are visible in the μCT images have been termed “tethers” (Lee et al., 2011), similar to those found in long bone growth plates (Chen et al., 2009). These tethers are relatively widespread by P28 (Figure 7), hence we had expected that growth of the basisphenoid would cease after this time point. However, the basisphenoid continues to increase in length significantly until P56, indicating that the observed mineralization does not necessitate a reduction in growth. This can be explained in part because, histologically, the radiopaque areas in the SOS correspond to altered cellular morphology and matrix staining in the resting zone and not bony bridging (Figure 9). These changes are indicative of cartilage mineralization as opposed to ossification and this distinction is important to make because it clearly affects the growth capacity of the structure (Chen et al., 2009; Lee et al., 2011). The role of cells within the resting zone, particularly as a reservoir for cells that constitute the proliferative zone, has been debated (Brighton, 1987; Lupu et al., 2001). Studies in growth plates of long bones have suggested the presence of a pre-proliferative zone (between the resting and proliferative zones), which can supply cells for continued growth (Abad et al., 2002). Therefore, the SOS could continue to grow in spite of tethered central resting zone. Additionally, the observed cellular morphology of the proliferative zone of the SOS between P28 and P76 (Figures 9J,K, open arrows showing rounding of cells) reflects an increase in extracellular matrix production or/and an increase in chondrocyte volume, both of which could facilitate growth (via hypertrophy) despite the prevalent mineralization at the resting zone. Similar growth changes have been described in the mouse nasal septal cartilage, where continued dimensional growth can be found although chondrocyte proliferation is decreasing from P5 to P15 (Wealthall and Herring, 2006).

While similar in their cellular structure, the ISS and SOS display significantly different patterns of mineralization (Figure 8), suggesting that independent regulatory mechanisms may be operating at each location. Chondrocytes populating the ISS (and all regions rostral to it) originate completely from neural crest cells, while the SOS has a dual origin: the rostral portion derived from neural crest cells and the caudal portion from mesoderm (McBratney-Owen et al., 2008). However, by P10, all cells of neural crest origin are thought to incorporate into the caudal portion of the rapidly growing presphenoid bone, leaving only cells of mesodermal origin at the SOS (McBratney-Owen et al., 2008). Hence the embryonic origins of the cells that give rise to each structure in the cranial base may be one factor influencing cellular activity and the subsequent timing or pattern of mineralization.

The rate of mineralization at the ISS and SOS in CD-1 mice (Wealthall and Herring, 2006), between P10 and P15 (~40–50 μm/day), is very similar to the rate of growth of the presphenoid and basisphenoid bones in P14 C57BL/6J mice studied here (~30–70 μm/day, Figure 5C). However, despite similarities between CD-1 mice and C57BL/6J mice, strain specific growth differences can be substantial and should not be discounted. For example, in the DBA/2J strain, ISS closure can be found as early as P10 (Adams et al., 2013). On-going studies in our lab indicate that the A/J and C57BL/6J mice differ in their timing of EO-BOS fusion, which can in turn, affect craniofacial dimensions. Such intraspecific (and indeed interspecific) heterochrony necessitates a thorough investigation into the postnatal ontogeny of different inbred strains (MacDonald and Hall, 2001).

The cranial base has long been considered a prominent factor contributing to the overall forward projection of the upper jaw (Hallgrímsson et al., 2007; Parsons et al., 2015). In this regard, we found that the presphenoid, along with the maxilla and zygoma, follows a cyclic growth pattern similar to the upper jaw (Figure 5C, right graph). This observation is intriguing since the presphenoid is part of the cranial base and traditionally viewed as being under different growth controls to the facial skeleton. Furthermore, the presphenoid shows a rapid increase in length up to P28 (Figure 3C, circled CC), however, minimal growth can be seen beyond P7 in the anterior part of the cranial base (Figure 3, circled Z). Hence, it can be derived that the A-P growth of the presphenoid (Figure 3C, circled CC) occurs primarily at the ISS and not at the sphenoethmoidal junction. Ma and Lozanoff (1996) in their assessment of the cranial base of Brachyrrhine (*Br/*+) mice, also concluded that growth deficiencies in the presphenoidal region contributes to the observed midface hypoplasia (Ma and Lozanoff, 1996). Parsons et al. (2015) used neonatal and adult mice with targeted cartilage growth disruption within the cranial base synchondroses (overgrowth model: *Pten*^*fl*∕*fl*^ Col2a1Cre and undergrowth models: *Trsp*^*fl*∕*fl*^ Col2a1Cre and *Papps2* mutation) and demonstrated coordinated changes between the cranial base and facial skeleton (midface) (Parsons et al., 2015). Together, these findings support the important and central role of the cranial base and its integration with facial growth.

**Figure 11:**
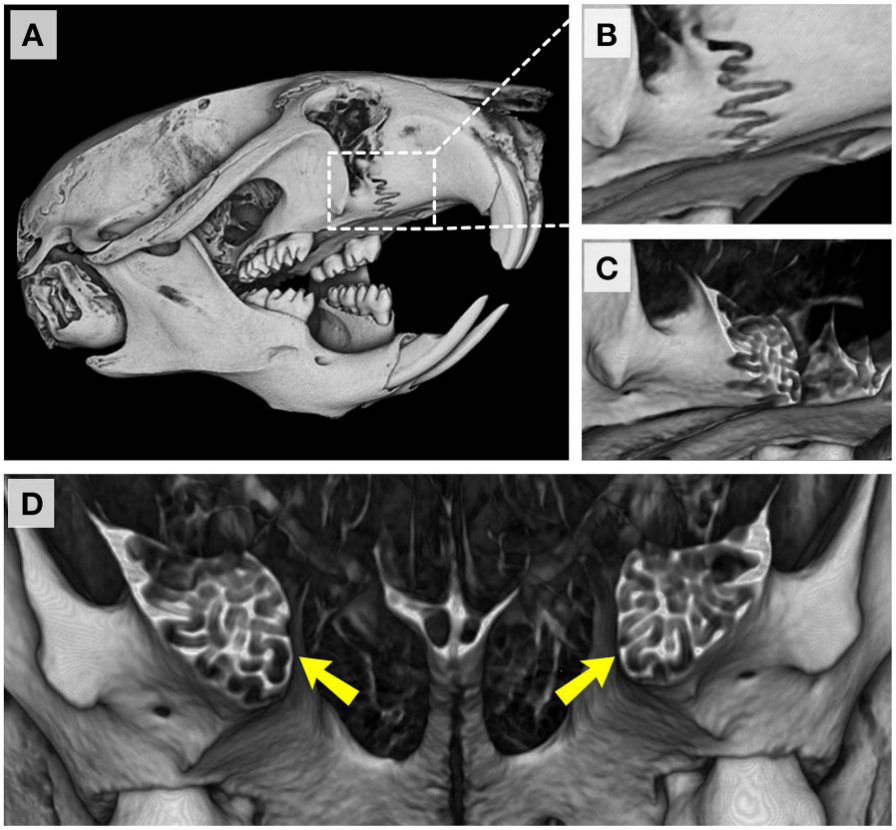
**Premaxillary-maxillary suture in the adult C57BL/6J mouse**. **(A)** Right fronto-lateral view of reconstructed micro-CT scans of a P42 mouse skull. **(B)** Magnified view of the right premaxillary-maxillary suture. **(C,D)** Coronal sections through the premaxillary-maxillary sutures showing the complexity of interdigitating bones at this site (yellow arrowheads).

Our focus on the cranial base and ontological changes from postnatal day 7 onwards is not meant to imply that growth at facial and cranial sutures is not important or that cranioskeletal growth during late gestational and neonatal ages is not relevant. With respect to the former, we have not addressed the normal timing of closure of the facial sutures here due to the characteristic complex interdigitation of apposing bone fronts as depicted in the example of the premaxillary-maxillary suture shown in Figure 11. Understanding the growth and activity at these sutures is of significant clinical interest since early obliteration of the facial sutures has been reported in many patients, including those with craniosynostosis syndromes, as well as in mouse models of many craniofacial anomalies (Perlyn et al., 2006; Cunningham et al., 2007; Purushothaman et al., 2011; Nah et al., 2012). The importance of precisely characterizing morphological, cellular, and molecular events occurring at each craniofacial growth site (sutures, synchondroses, condylar cartilage) cannot be overstated. Consequently, future efforts will be aimed at describing temporal changes at the multiple facial sutures and how they each contribute, along with the cranial base, to overall postnatal facial projection. Furthermore, although the data presented in this report will support investigations of animal models of common craniofacial dysmorphologies, integrating morphometric data from late gestational and neonatal ages will be necessary to fully understand the ontogeny of more severe craniofacial malformations.

## Author contributions

SV, EC, and TC conceived the study. SV and EC performed scans and landmarking, and SV did the histology and morphometric analyses. SV and TC wrote the manuscript, with EC contributing to editing.

### Conflict of interest statement

The authors declare that the research was conducted in the absence of any commercial or financial relationships that could be construed as a potential conflict of interest.
