# Supplementary material for: Postnatal Ontogeny of the Cranial Base and Craniofacial Skeleton in Male C57BL/6J Mice: A Reference Standard for Quantitative Analysis
Source: Front Physiol. 2016 Jan 12;6:417. doi: 10.3389/fphys.2015.00417 (PMC4709510; doi:10.3389/fphys.2015.00417)
Supplement: Supplementary file 1 [file SupplementaryData.pdf]

*Supplementary Material*

# Postnatal ontogeny of the cranial base and craniofacial skeleton in male C57BL/6J mice: A reference standard for quantitative analysis

**Siddharth R. Vora[1,2,4], Esra D. Camci[1,4] & Timothy C. Cox[3,4,5] ***

*** Correspondence:**
Prof Timothy C. Cox
Center for Developmental Biology & Regenerative Medicine
Seattle Children's Research Institute
M/S C9S-5
1900 9th Avenue
Seattle, WA, 98101, USA
tccox@uw.edu

**Supplementary Figure 1. Landmarks used in this study (shown on a P56 skull).** Descriptions of landmarks are provided in Supplementary Table 1.

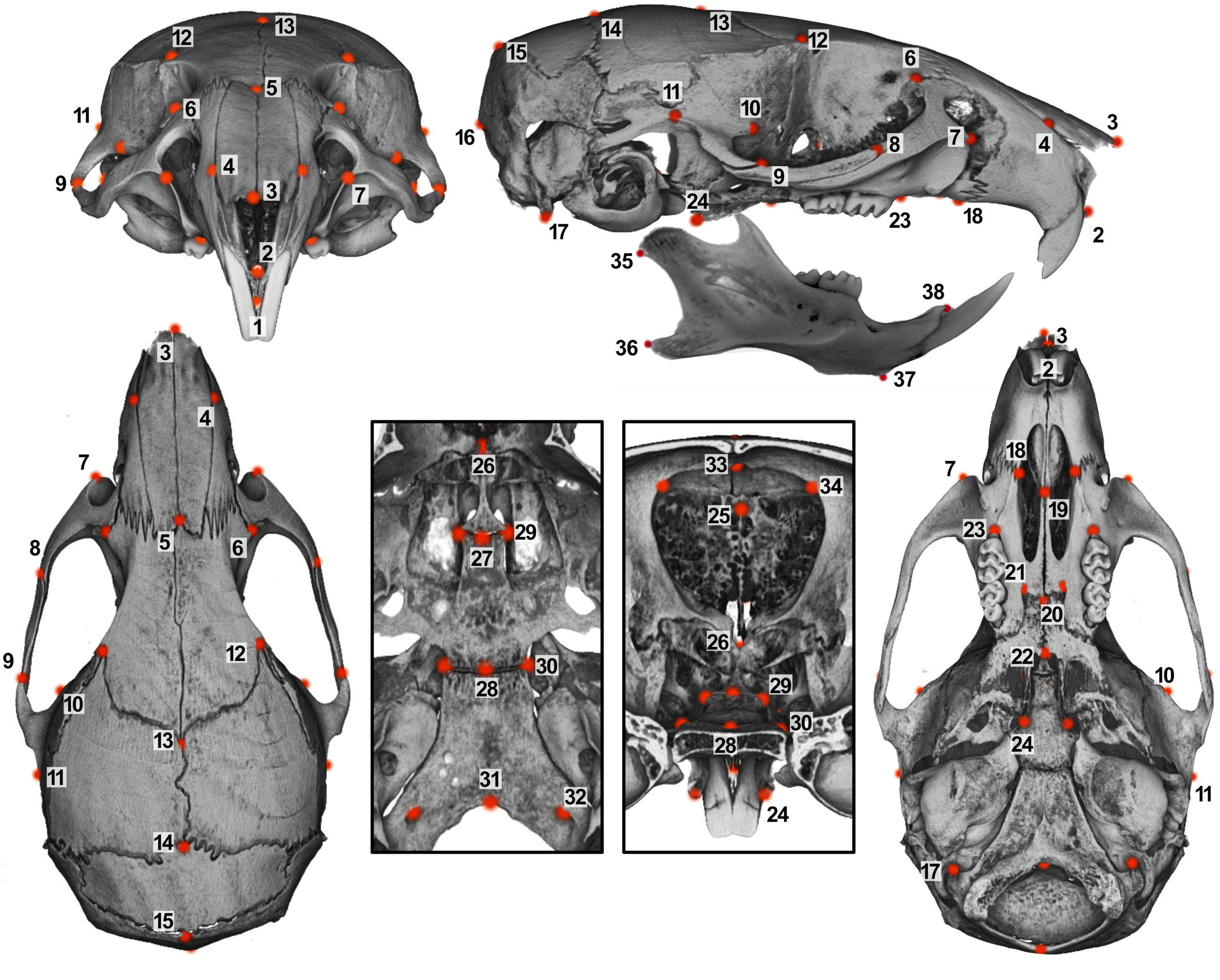

**Supplementary Figure 2. Measurement of plane-projected distances for analysis of antero-posterior growth.**

Micro-CT scan of a representative P28 animal with constructed mid-sagittal and transverse planes (**top**), with a slice view through the mid-sagittal plane (**bottom**). Landmarks #1, #18 (right), #20 and #22, which are used to measure the premaxillary, maxillary and palatal bones, are shown in red (see Supplementary Figure 1 and Supplementary Table 1). Parallel axial planes (perpendicular to the mid-sagittal and transverse planes) are constructed thought these landmarks and the distance between them measures the A-P dimension of the individual bones. For bilaterally paired landmarks (e.g. #18 shown here), an average of the right and left measurement is for analysis.

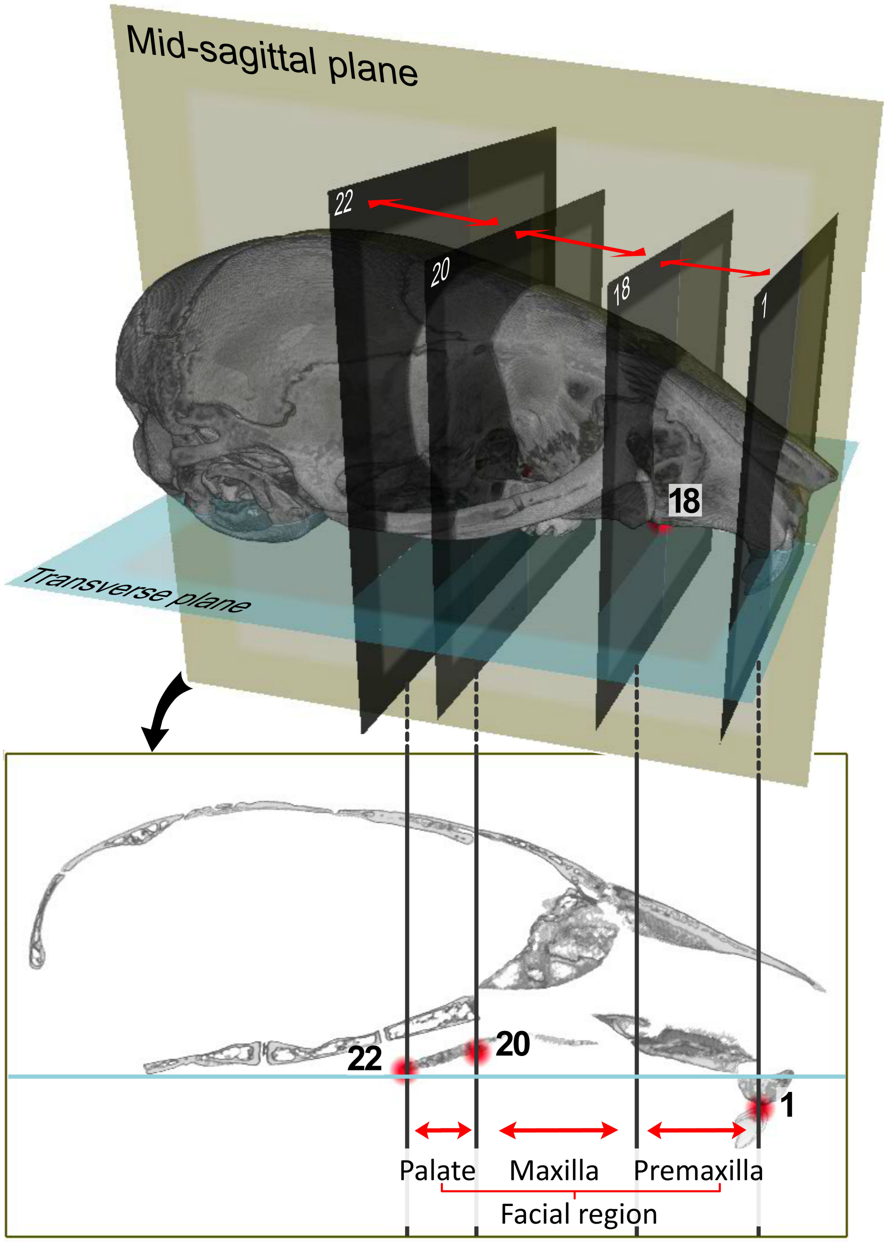

**Supplementary Table 1. List of landmarks used to derive craniofacial measurement.**
Mouse skull with annotated landmarks is shown in Supplementary Figure 1.

| # | Description | Type |
|---|---|---|
| **1** | Midpoint mesial to maxillary incisors | Single |
| **2** | Anterior point at base of nasal aperture | Single |
| **3** | Nasale | Single |
| **4** | Anterior point on intersection of premaxillary and nasal bones | Paired |
| **5** | Nasion | Single |
| **6** | Intersection of maxilla, frontal and lacrimal bones | Paired |
| **7** | Anterior-superior point on zygomatic process of maxilla (lateral to infra orbital fissure) | Paired |
| **8** | Anterior end of zygomatic bone | Paired |
| **9** | Posterior end of zygomatic bone | Paired |
| **10** | Anterior point on base of zygomatic process of temporal bone | Paired |
| **11** | Posterior point on base of zygomatic process of temporal bone | Paired |
| **12** | Most prominent lateral point on frontal-parietal suture | Paired |
| **13** | Bregma | Single |
| **14** | Lamda | Single |
| **15** | Mid-sagittal point of intersection of interparietal and occipital bones | Single |
| **16** | Ophisthion | Single |
| **17** | Lateral point on mastoid process | Paired |
| **18** | Lateral premaxillary - maxillary suture | Paired |
| **19** | Medial premaxillary - maxillary junction | Single |
| **20** | Midline point on maxillary-palatal suture | Single |
| **21** | Anterior point on palatine foramen | Paired |
| **22** | Posterior point on palate | Single |
| **23** | Crest of alveolar process (mesial to 1st molar) | Paired |
| **24** | Posterior point on pterygoid process | Paired |
| **25** | Spine of ethmoidal crest | Single |
| **26** | Anterior point on midline of presphenoid (junction with ethmoid) | Single |
| **27** | Intesphenoidal synchondrosis (mid-point of endocranial rostral margin of basisphenoid) | Single |
| **28** | Spheno-occipital synchondrosis (mid-point of endocranial caudal margin of basisphenoid) | Single |
| **29** | Lateral point of endocranial rostral margin of basisphenoid | Paired |
| **30** | Lateral point of endocranial caudal margin of basisphenoid | Paired |
| **31** | Basion | Single |
| **32** | Anterior-medial point on hypoglossal canal | Paired |
| **33** | Mid-point on interior crest of frontal bone | Single |
| **34** | Superior-lateral point on interior crest of frontal bone | Paired |
| **35** | Posterior inferior point on mandibular condyle | Single |
| **36** | Posterior tip of the angular process | Single |
| **37** | Anterior inferior most point on the body of the mandible | Single |
| **38** | Midpoint on alveolar bone lingual to mandibular incisor | Single |
